# Are dietary intake and nutritional status of specific polyunsaturated fatty acids correlated with sarcopenia outcomes in community-dwelling older adults with sarcopenia? – Exploratory results from ENHANce

**DOI:** 10.1186/s12877-023-04007-9

**Published:** 2023-05-05

**Authors:** Jolan Dupont, Eva Wauters, Lenore Dedeyne, Laura Vercauteren, Nadjia Amini, Laurence Lapauw, Christophe Matthys, Sabine Verschueren, Jos Tournoy, Katrien Koppo, Evelien Gielen

**Affiliations:** 1 Geriatrics & Gerontology, Department of Public Health and Primary Care, KU Leuven, Leuven, Belgium; 2 Department of Geriatric Medicine, University Hospitals Leuven, Herestraat 49, B-3000 Leuven, Belgium; 3 Clinical and Experimental Endocrinology, Department of Chronic Diseases and Metabolism, KU Leuven, Leuven, Belgium; 4 Department of Endocrinology, University Hospitals Leuven, Leuven, Belgium; 5 Research Group for Musculoskeletal Rehabilitation, Department of Rehabillitation Sciences, KU Leuven, Leuven, Belgium; 6 Exercise Physiology Research Group, Department of Movement Sciences, KU Leuven, Leuven, Belgium

**Keywords:** Sarcopenia, Older adults, Polyunsaturated fatty acids, Omega-3, Omega-6

## Abstract

**Aims:**

To explore the relationship between dietary polyunsaturated fatty acids (PUFAs) intake, nutritional PUFAs status and sarcopenia outcomes in sarcopenic older adults.

**Methods:**

The Exercise and Nutrition for Healthy AgeiNg (ENHANce) is an ongoing 5-armed triple blinded randomized controlled trial, in sarcopenic older adults (> 65y) aiming to assess the effect of combined anabolic interventions (protein, omega-3 supplement and exercise) on physical performance in these adults, compared to single/placebo interventions. Baseline data were used for a secondary, exploratory, cross-sectional analysis. Dietary PUFAs intake was assessed with 4-day food records, status with RBC membrane fatty acids profiles. Spearman’s rho(ρ) correlation coefficients were calculated to explore associations of PUFAs intake and status with sarcopenia-defining parameters (muscle strength, mass and physical performance), physical activity (step count) and quality of life (SF-36, SarQoL).

**Results:**

In total, 29 subjects (9♂/20♀, mean age 76.3 ± 5.4y) were included. Total omega-3 intake of participants (1.99 ± 0.99 g/d) was below the recommended intake (♂:2.8–5.6 g/d; ♀:2.2–4.4 g/d). Intake and status of PUFAs were not correlated. Regarding correlations with outcomes, α-linolenic acid status was inversely associated with appendicular lean mass (aLM) (ρ:-0.439; *p* = 0.017), whereas docosahexaenoic acid status was positively associated with aLM (ρ:0.388; *p* = 0.038). Some omega-3 PUFAs intake and status markers were positively associated with step count, SF-36 and SarQoL scores, whereas gamma-linolenic acid status was inversely associated with SF-36 physical component summary score (ρ = -0.426; *p* = 0.024).

**Conclusions:**

Although intake of omega-3 and omega-6 was low, the present exploratory study generated new hypotheses for potential correlations of PUFAs intake and status with sarcopenia outcomes in older adults with sarcopenia.

**Supplementary Information:**

The online version contains supplementary material available at 10.1186/s12877-023-04007-9.

## Introduction

Diet plays an important role in the development and treatment of sarcopenia, the age-related loss of muscle mass and function [1]. Primary sarcopenia is age-driven and is a growing concern in the ageing population. It can seriously affect the daily life of an older adult, e.g., by limiting the ability to lift heavy objects like groceries or through difficulties to climb the stairs or to walk [2]. Sarcopenia affects up to 29% of community-dwelling older adults and up to 33% of nursing home residents [3]. Malnutrition and low protein intake contribute to sarcopenia onset or progression [4], whereas nutritional supplementation (alone or combined with exercise) is currently the recommended treatment strategy for sarcopenia [5, 6]. Besides protein intake, the intake of polyunsaturated fatty acids (PUFAs) is also suggested to influence muscle physiology and sarcopenia progression [7, 8].

Dietary fat intake is a major determinant of muscle structure and function. Fatty acids can act as an energy source for the muscle, but are also incorporated in various structures of the skeletal muscle containing phospholipid membranes (e.g., sarcolemma or mitochondria), thus optimizing the muscle’s structure and function [9–11]. In general, omega-3 PUFAs are suggested to have beneficial effects on the muscle, whereas omega-6 PUFAs might have negative effects on the muscle through their pro-inflammatory properties [12]. Therefore, a higher omega-3 to omega-6 PUFAs dietary intake ratio is considered beneficial for musculoskeletal health [13, 14], as well as other health domains like cardiovascular health [15]. In this context, a high omega-3 PUFAs intake is gaining increasing attention as a potential treatment for sarcopenia [16]. Increasing intake of omega-3 PUFAs might counteract or prevent sarcopenia through some of its properties that are potentially beneficial for the muscle (e.g., improving muscle protein synthesis through its anti-inflammatory properties [16, 17], reducing anabolic resistance [11, 16, 18] and reducing insulin resistance [19]).

Detailed data on usual dietary PUFAs intake in well-defined sarcopenic older adults are lacking. For this purpose, food records are a useful tool to measure dietary intake but carry an inherent risk of bias due to recall problems or socially desirable responses [20]. Moreover, this might be a measure reflecting only short-term intake and not how this intake interferes with nutritional status. An adequate intake should ideally lead to an optimal nutritional status. Thus, it might be interesting to not only assess intake, but also status. Therefore, an objective biomarker for PUFAs status, such as the fatty acids composition of the red blood cell (RBC) membrane, is useful [21, 22], as this represents the nutritional PUFAs status based on the intake over the past month or two [23]. This longer-term marker of PUFAs status has been reported in several populations, e.g. children [24], postmenopausal women [25] or men with prostate cancer [26] but not yet in sarcopenic older adults.

Therefore, the aim of the present study was to assess the dietary intake of PUFAs – through food records – and PUFAs status – through RBC membrane fatty acids composition – in a sample of well-defined sarcopenic older adults according to the recent European Working Group on Sarcopenia in Older People (EWGSOP2) criteria. As the first study to do so in this population, we also aimed to determine the correlation between intake and status of PUFAs. Furthermore, the present study intended to explore associations between dietary intake and nutritional status of PUFAs and individual sarcopenia-defining parameters (muscle strength, muscle mass and physical performance) as well as physical activity and quality of life (QoL). We hypothesized that intake of omega-3 PUFAs is low in sarcopenic older adults and that intake is well correlated with status. Moreover, we presumed that higher intake of omega-3 is associated with improved muscle strength and mass, as well as higher QoL scores and more physical activity.

## Methods

### Subjects and study design

A secondary, exploratory, cross-sectional analysis was performed with baseline data of the ongoing Exercise and Nutrition for Healthy AgeiNg study (ENHANce) [27]. Older adults (aged 65 years or older) with sarcopenia were recruited for ENHANce in the University Hospitals Leuven, Belgium. ENHANce is a 5-armed triple-blinded randomized controlled trial (RCT) with the aim to assess the effect of combined anabolic interventions (protein, omega-3 supplement and physical exercise) on physical performance in older adults with sarcopenia, compared to single interventions or placebo. Methods are described in detail elsewhere [27]. In short, community-dwelling or assisted living older adults (> 65y) were recruited to participate in a 12-week intervention, followed by 12-weeks of follow-up. At screening visit, sarcopenia status was assessed. In the present analysis, only participants suffering from probable/confirmed/severe sarcopenia, according to the EWGSOP2, with complete baseline data and food records were eligible for inclusion [1]. Participants were included from February 2018 until May 2021. Participants were excluded if one of the following was present: not able to communicate in Dutch, English or French; allergy to milk, soy, peanut or peanut oil; Mini-Mental State Examination (MMSE) score < 21 [28]; terminal illness with a prognosis less than 6 months; a protein intake higher than 1.5 g/kg body weight (BW)/day; participation in a training program more or equal than twice per week for the last 6 months; active uncontrolled disease or acute cardiovascular problems (or negative advise of doctor to perform physical activities); 25-hydroxyvitamin D blood concentration < 20 ng/L; glomerular filtration rate < 30 ml/min/1.73m^2^; fasting glycaemia > 126 mg/dl; usage of anti-diabetic medication; presence of impairments or diseases that impose problems to study participation. After inclusion, the participants were randomized into one of the five intervention arms. The study is registered at ClinicalTrials.gov (NCT03649698). Ethical approval was obtained by the UZ/KU Leuven Ethical committee (s60763). All subjects provided written informed consent. The study was reported according to the STROBE-nut checklist [29], see Supplementary Table S1.

### Sarcopenia-defining parameters

Muscle strength was evaluated by measuring grip strength with the Jamar 1 hand-held dynamometer (TEC Inc., Clifton, NJ, USA). Maximal grip strength was recorded as the highest of three measurements at both sides [30]. Lower extremity muscle strength was measured through the chair stand test [1], in which the time that a participant needs to stand up and sit again for five consecutive times is registered. Muscle mass was assessed through appendicular lean mass (aLM). For this purpose, subjects received a whole-body Dual-energy X-ray absorptiometry (DXA) scan on a QDR 4500A Discovery scanner (Hologic Inc, Bedford, MA, USA). Scans were analysed using Hologic APEX 4.0 software. Physical performance was assessed by gait speed, expressed as meters per second (m/s). The subject was instructed to walk six meters at usual pace. The time was measured over four meters, i.e. starting from the moment the participant’s foot passed the mark of the one meter line until the foot passed the mark of the five meter line according to the British Columbia (BC) guidelines [31]. The Short Physical Performance Battery (SPPB), a multicomponent test combining a chair stand test, gait speed and a balance test, was also used to assess physical performance. Each individual domain is scored from 0–4 and the total score is 0–12 points, with a score of 12 indicating best physical performance [32].

### Sarcopenia definition

If handgrip strength was low (< 27 kg for men or < 16 kg for women) or chair stand test time was > 15 s, muscle strength was considered low and the subject was categorized as having probable sarcopenia [1]. When, additionally, muscle mass was low (SMI < 7.0 kg/m^2^ for men or < 5.5 kg/m^2^ for women), the subject had confirmed sarcopenia. In case that gait speed was also low (< 0.8 m/s), sarcopenia was considered severe. Subjects with probable, confirmed, or severe sarcopenia were included in this analysis.

### Dietary PUFAs intake through 4-day food records

All included subjects were asked to report their daily food intake by using 4-day food records that were provided at the screening visit of ENHANce. Subjects were instructed to complete four food records on non-consecutive days – including three weekdays and one weekend day – spread over a period of 2 weeks. Participants were asked not to adapt their usual diet and to register the specific food items and portions as accurately as possible (in grams or household measures). In order to avoid missing data and to optimize quality, food records were reviewed together with the participant upon submission. Subsequently, food records were examined in detail by a member of the research team using the nutrients described from the Belgian Food Composition Database (FCDB) NUBEL [33]. For the food components or meals that were not included in the NUBEL database, a protocol was created to translate these food components or meals into standard food components present in the NUBEL databank. Homemade meals without a match in the NUBEL database were converted into ingredients using standard recipes described in ‘Ons Kookboek’ [34]. For packed food or prepared food, the nutritional information on the food label was used, if available. When portion sizes were defined in (household) measurements (e.g., “a spoon of”, “a cup of”, “a slice of”, “a piece of”), these were converted into grams using the ‘Maten en Gewichten’ version of 2005 by the Belgian Superior Health Council [35]. When portion sizes were not mentioned, a standard portion size was assumed as defined in the ‘Maten en Gewichten’ database [35]. In the current study, a specific focus was on PUFAs. Therefore, if the specific PUFA values were missing in the Belgian FCDB, alternative FCDBs like (in order of importance) the Finnish (Fineli), the FoodData Central (U.S. Department of Agriculture), InterNubel and the Dutch “Nederlands Voedingsstoffenbestand” (NEVO) were used to complete missing data [36–39]. In case that details on PUFAs’ nutrient values of an ingredient could not be found in one of the previously mentioned databases, absence of PUFAs in that particular ingredient was presumed. In case omega-3 supplements were known to be used, these were taken into account. Average daily intake of total energy, total carbohydrates, total fat, protein, omega-3 PUFAs (Total omega-3 PUFAs, eicosapentaenoic acid [EPA]; docosahexaenoic acid [DHA], EPA + DHA and α-Linolenic acid [ALA]) and omega-6 PUFAs (Total omega-6 PUFAs and Linoleic acid [LA]) were calculated by averaging the intake over the four recorded days. Macronutrient intake of fat, protein and carbohydrates was converted into Energy % (E%) by using the Atwater system (fat = 9 kcal/g; carbohydrates = 4 kcal/g and protein = 4 kcal/g) [40]. Recommended dietary intake thresholds were used as recommended by the Belgian Superior Health Council (HGR) [41].

### Nutritional PUFAs status through RBC fatty acid composition

Venous blood samples were collected by a phlebotomist. Samples were centrifuged immediately for 10 min at 1500 × g at 4 °C and RBC were separated from the blood sample. A solution of phosphate buffered solution, containing 1.5 mg/mL ethylenediaminetetraacetic acid, was added to preserve RBC. Samples were stored at -80 °C until in batch analysis. RBC fatty acid composition was analysed by OmegaQuant (South Dakota, U.S.A.), using gas chromatography (GC) with flame ionization detection [15]. RBC was transferred to a screw-cap glass vial and 14% boron trifluoride (Sigma-Aldrich, St. Louis, MO) and hexane (EMD Chemicals, USA) was added. The vial was briefly vortexed and heated in a hot bath at 100˚C for 10 min. After cooling, HPLC grade water was added, the tubes were recapped, vortexed and centrifuged to help separate layers. An aliquot of the hexane layer was transferred to a GC vial. GC was carried out using a GC-2010 Gas Chromatograph (Shimadzu Corporation, Columbia, MD) equipped with a SP-2560, 100-m fused silica capillary column (0.25 mm internal diameter, 0.2 um film thickness; Supelco, Bellefonte, PA). Fatty acids were identified by comparison with a standard mixture of fatty acids (GLC OQ-A, NuCheck Prep, Elysian, MN) which was also used to determine individual fatty acid calibration curves. For present analysis, data of cis n-3 polyunsaturated fatty acids 18:3 (ALA), 20:5 (EPA), 22:5 (Docosapentaenoic acid or DPA) and 22:6 (DHA) were used. Fatty acid composition was expressed as a percentage of total identified fatty acids. Omega-3 index (O3I) was calculated as the sum of EPA and DHA content of red blood cells membranes, expressed as a percent of total identified fatty acids [15]. Similarly, data on cis n-6 polyunsaturated fatty acids 18:2 (LA), 18:3 (Gamma-linolenic acid or GLA), 20:3 (Dihomo-gamma-linolenic acid or DGLA) and 20:4 (Arachidonic acid or AA) were used.

### QoL and physical activity

Two health-related QoL instruments were used, namely the Short Form - 36 Health Survey (SF-36) and the Sarcopenia Quality of Life questionnaire (SarQoL). SF-36 is a generic health-related QoL instrument that includes eight health domains: physical functioning, role limitations due to physical and emotional health, mental health, bodily pain, general health, vitality and social functioning [42]. These items make use of a norm-based scoring method, combining these eight health domains into component summary scales scores for mental and physical QoL. Summary scores – standardised to a reference population – were calculated using the STATA module “sf36”, as described elsewhere [43], with higher scores suggesting better QoL. SarQoL is a specific health-related QoL questionnaire designed and validated for use in older adults with sarcopenia, assessing seven health domains: physical and mental health, locomotion, body composition, functionality, activities of daily living, leisure activities and fears. These domains are combined into a total SarQoL score, ranging from 0–100 with a higher score indicating better QoL [44].

Physical activity was assessed through the Dynaport MoveMonitor + (McRoberts, The Netherlands), with build-in tri-axial accelerometer. This method was validated for use in community-dwelling older adults [45]. Subjects were instructed to wear this device on five consecutive days prior to baseline visit. If worn less, data were excluded from the analysis. Total steps per day were calculated using the device software of the manufacturer.

### Statistics

Descriptive statistics were used to summarize subjects’ baseline characteristics. Normality was checked through the Shapiro-Wilk test. Gender differences were evaluated through a two-sample t-test, with a *p*-value < 0.05 considered significant. Coefficient of variation (CV) was calculated as mean/standard deviation *100. Correlations were assessed through pairwise calculation of the Spearman’s rank correlation (ρ). Due to the exploratory nature of the study, no correction for multiple testing was applied. A correlation was considered significant if *p*-value < 0.05. The correlation coefficient (ρ) was interpreted as poor if < 0.3, fair if ≥ 0.3 and < 0.6, moderate if ≥ 0.6 and < 0.8 and very strong if ≥ 0.8 [46]. Results were presented in a heat plot. Statistics were performed using STATA SE 16.1.

## Results

### Subjects

A total of 29 community-dwelling older adults (20 women and 9 men) with probable, confirmed or severe sarcopenia were included. Baseline characteristics are described in Table 1. Mean age of the participants was 76.3 ± 5.4 years. According to EWGSOP2 criteria, 12 persons were categorized as having probable sarcopenia, 16 confirmed sarcopenia and one person was considered severely sarcopenic. In 6 out of 29 subjects, data on SarQoL questionnaire were missing or incomplete and 6 participants had incomplete data on the physical activity monitor. As such, these participants were excluded from the analyses with SarQoL and physical activity parameters, respectively.

**Table 1:** Baseline characteristics

Variable	Mean (SD)		n
Age (years)	76.3 (5.4)	29	
Number of Male/Female subjects	9/20	29	
Weight (kg)	70.98 (15.86)	29	
Body Mass Index (kg/m^2^)	25.3 (4.8)	29	
Chair stand test time (s)	20.1 (4.9)	28	
Handgrip strength (kg)	25.6 (9.1)	29	
Appendicular Lean Mass [ALM] (kg)	16.64 (3.87)	29	
Gait speed (m/s)	1.01 (0.17)	29	
Short Physical Performance Battery score [SPPB]	8.4 (1.7)	29	
Number of steps/day	6879.6 (5721.5)	23	
SF-36 Physical component summary score	40.9 (8.4)	29	
SF-36 Mental component summary score	50.2 (10.2)	29	
SarQoL total score	61.6 (15.3)	23	
Sarcopenia status (n)			
Probable sarcopenia		12	
Confirmed sarcopenia		16	
Severe Sarcopenia		1	

### PUFAs intake and status in sarcopenic older adults

Detailed summary of the participants’ intake and status of PUFAs can be found in Table 2. Total energy intake was significantly higher in men (2086.47 ± 279 kcal/d) compared to women (1808.45 ± 336.31 kcal/d). Furthermore, RBC %GLA was slightly higher (*p* = 0.044) in women (0.10 ± 0.03%) compared to men (0.07 ± 0.04%). Otherwise, no other significant gender differences were found.

**Table 2:** Dietary intake and RBC membrane composition of sarcopenic older adults

Variable	All participants	Women	Men	*P*-value	CV (%)	
	Mean (SD) Median [IQR]	Mean (SD) Median [IQR]	Mean (SD) Median [IQR]			
	*N* = 29	*N* = 20	*N* = 9			
***Dietary intake through food records***						Recommended dietary intake [41]
Energy (kcal/d)	1894.73 (340.81)	1808.45 (336.31)	2086.47 (279.19)	0.040^*^	17.99	♀&♂:30 kcal/kg body weight/d
Protein (E%)	14.83 [13.45–17.01]	15.35[13.55–17.67]	14.71 [13.45–15.30]	0.306	19.67	♀&♂: > 15%
Total carbohydrate (E%)	46.01 [38.64–48.15]	46.56 [39.09–48.72]	45.94 [38.64–46.06]	0.905	14.84	♀&♂: 50–55%
Total fat (E%)	34.34 (5.67)	34.55 (5.75)	33.86 (5.80)	0.769	16.50	♀&♂: > 20% but < 30–35%
Saturated fatty acids (g/d)	25.55 [21.29–37.16]	24.76 [20.38–33.21]	36.17 [23.48–38.20]	0.248	37.20	♀: < 22 g/d; ♂: < 28 g/d
Monounsaturated fatty acids [MUFA’s] (g/d)	23.76 [20.10–31.19]	22.47 [19.18–28.92]	26.94 [23.17–32.72]	0.724	35.34	♀:22-44 g/d; ♂:28-55 g/d
Polyunsaturated fatty acids [PUFA’s] (g/d)	10.44 [8.52–14.41]	9.92 [7.88–14.65]	11.61 [8.52–13.59]	0.773	43.91	♀:11-22 g/d; ♂:14-28 g/d
*Omega-3 PUFAs*						
Total Omega-3 (g/d)	1.99 (0.99)	1.94 (0.91)	2.11 (1.21)	0.675	49.77	♀: 2.2–4.4 g/d; ♂: 2.8–5.6 g/d
ALA (g/d)	1.62 (0.90)	1.61 (0.84)	1.65 (1.07)	0.920	55.28	♀: 2.2 g/d; ♂: 2.8 g/d
EPA (g/d)	0.03 [0.02–0.15]	0.03 [0.02–0.09]	0.12 [0.03–0.25]	0.083	117.21	
DHA (g/d)	0.08 [0.04–0.16]	0.08 [0.04–0.13]	0.12 [0.07–0.24]	0.182	82.41	
Total EPA + DHA (g/d)	0.11 [0.07–0.36]	0.11 [0.06–0.26]	0.27 [0.08–0.42]	0.104	93.66	♀&♂: 0.250–0.500 g/d
*Omega-6 PUFAs*						
Total Omega-6 (g/d)	8.80 [6.16–11.34]	8.82 [6.05–11.39]	7.38 [6.55–10.64]	0.950	46.85	♀: 8.8–18.0 g/d; ♂: 11.0–22.0 g/d
Total LA (g/d)	7.60 [5.96–10.49]	7.74 [5.61–10.34]	6.90 [6.55–10.49]	0.965	48.90	♀: 8.8 g/d; ♂: 11.0 g/d
***PUFAs status through RBC fatty acids composition***						Laboratory reference values [47]
*Omega-3 PUFAs*						
RBC ALA (%)	0.18 (0.05)	0.19 (0.06)	0.15 (0.04)	0.078	31.00	0.08–0.40%
RBC DPA (%)	3.13 (0.36)	3.13 (0.33)	3.15 (0.46)	0.895	11.57	1.70–5.10%
RBC EPA (%)	1.05 [0.83–1.31]	1.00 [0.81–1.35]	1.13 [1.00–1.31]	0.636	34.94	0.29–4.30%
RBC DHA (%)	5.40 (0.85)	5.28 (0.96)	5.65 (0.48)	0.294	15.71	2.25–8.72%
RBC Omega-3 index	6.54 (1.12)	6.40 (1.25)	6.84 (0.70)	0.335	17.11	2.66–12.20
*Omega-6 PUFAs*						
RBC LA (%)	10.23 (1.61)	10.42 (1.87)	9.80 (0.70)	0.351	15.72	7.81–18.10%
RBC GLA (%)	0.09 (0.04)	0.10 (0.03)	0.07 (0.04)	0.044^*^	43.14	0.00–0.26%
RBC AA (%)	15.45 (1.16)	15.46 (1.24)	15.42 (1.05)	0.935	7.53	10.11–19.00%
RBC DGLA (%)	1.59 [1.47–2.01]	1.59 [1.40–1.99]	1.60 [1.50–2.02]	0.733	24.93	0.93–2.90%

Standard deviations in parentheses. *P*-values of two-sample t-test according to sex

*CV* Coefficient of variation, *IQR* Interquartile range, *RBC* Red blood cell, *MUFAs* Monounsaturated fatty acids, *PUFAs* Polyunsaturated fatty acids, *LA* Linoleic acid, *GLA* Gamma-linolenic acid, *AA* Arachidonic acid, *DGLA* Dihomo-gamma-linolenic, *ALA* α-Linolenic acid, *EPA* Eicosapentaenoic acid, *DPA* Docosapentaenoic acid, *DHA* Docosahexaenoic acid

^*^P < 0.05

When correlating intake with RBC status, correlation coefficients were small and no significant association between the RBC composition and food records of ALA, EPA, DHA and LA was found (Table 3). For GLA, DGLA, AA no trustworthy calculation through the food records was available, due to the limited availability of details regarding these PUFAs in the specific FCDB. As such, no comparison with RBC values was available.

**Table 3:** Correlations between PUFAs’ dietary intake and PUFAs status in RBC fatty acids composition

Food records intake	RBC fatty acids parameters	Spearman’s Rho (ρ)	*P*-value
Omega-3 PUFAs			
EPA intake	RBC %EPA	0.139	0.471
DHA intake	RBC %DHA	0.234	0.223
ALA intake	RBC %ALA	-0.046	0.812
Omega-6 PUFAs			
LA intake	RBC %LA	0.065	0.740

*RBC* Red blood cell, *PUFAs* Polyunsaturated fatty acids, *EPA* Eicosapentaenoic acid, *DHA* Docosahexaenoic acid, *LA* Linoleic acid

Mean recorded omega-3 intake was lower than the recommended intake by the Belgian Superior Health Council (HGR), nor did the mean or median of ALA, EPA and DHA reach the recommendation threshold [41]. Median omega-6 intake was just above the threshold for women, but not for men since they are recommended to have a higher intake, compared to women. LA intake covered most of the omega-6 intake, whereas ALA intake was the largest of all examined omega-3 PUFAs.

Regarding PUFAs status, all omega-3 and omega-6 status parameters in the RBC were within the laboratory reference suggested by the manufacturer, corresponding with a 99% confidence interval [47]. Of the omega-3 PUFAs, DHA was the largest contributor to the RBC membrane composition, whereas AA was the major omega-6.

### Sarcopenia-defining parameters

**Fig. 1:**
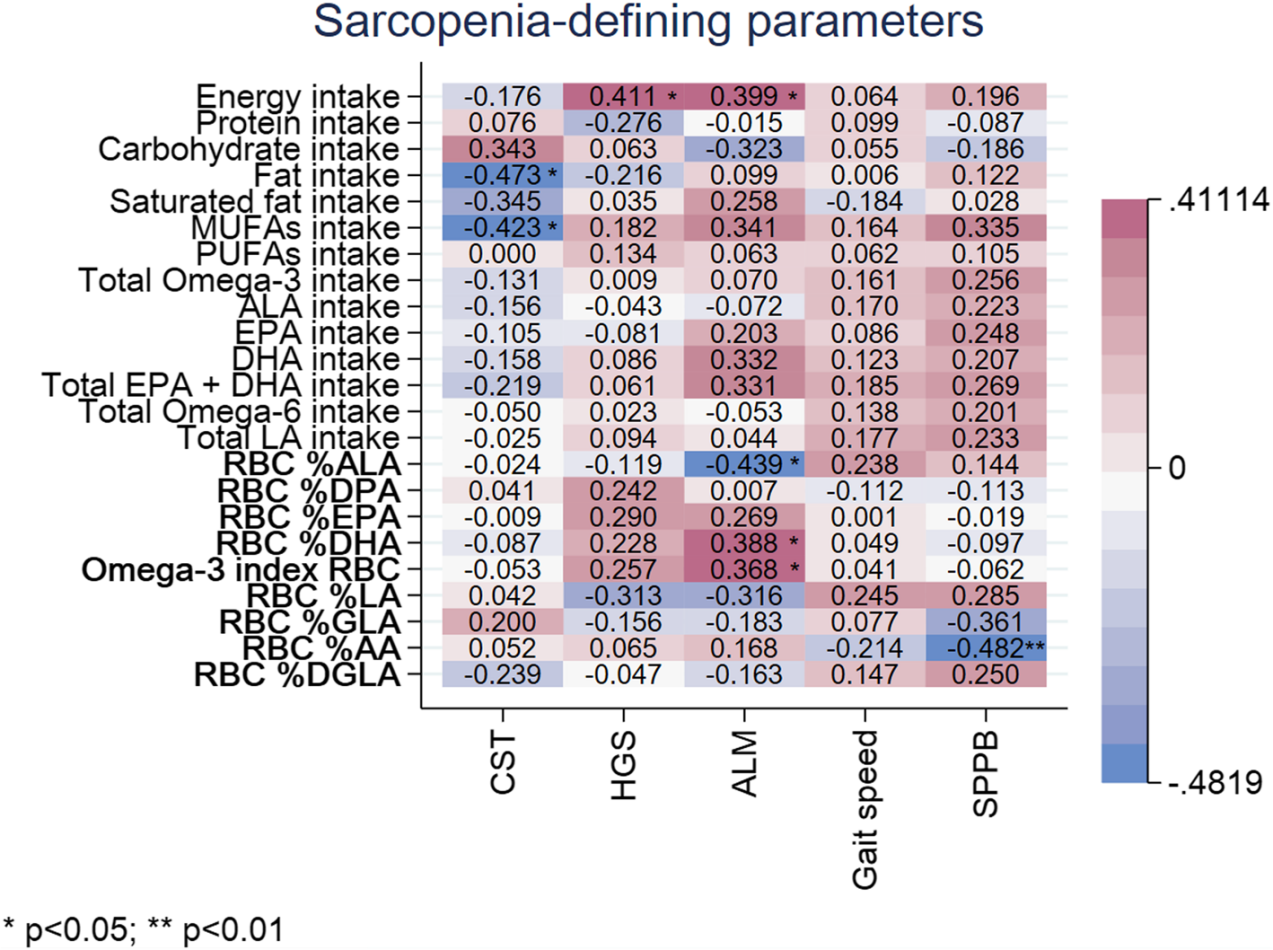
Heat plot representing correlation coefficients (ρ) between sarcopenia-defining parameters and PUFAs’ dietary intake and status. RBC = red blood cell; MUFAs = monounsaturated fatty acids; PUFAs = polyunsaturated fatty acids; LA = Linoleic acid; GLA = Gamma-linolenic acid; AA = Arachidonic acid; DGLA = Dihomo-gamma-linolenic; ALA = α-Linolenic acid; EPA = eicosapentaenoic acid; DPA = Docosapentaenoic acid; DHA = docosahexaenoic acid; CST = chair stand test; HGS = handgrip strength; aLM = appendicular lean mass; SPPB = Short Physical Performance Battery

Correlations of PUFAs intake and status with different sarcopenia-defining parameters (chair stand test, handgrip strength, aLM, gait speed and SPPB) are presented in Fig. 1. A summary of all examined correlations, can be found in Supplementary Data, Table S2.

No significant correlations between PUFAs intake and sarcopenia parameters were found. In contrast, omega-3 PUFAs status of ALA was found to have a fair but inverse correlation with aLM (ρ:-0.439; *p* = 0.017, suggesting that with increasing RBC %ALA, aLM was lower in our participants. However, both RBC %DHA and O3I had a fair but positive correlation with aLM (ρ:0.388; *p* = 0.038 for DHA and ρ:0.368; *p* = 0.049 for the O3I). Regarding omega-6 PUFAs status, RBC %AA was fair but inversely correlated with the SPPB score (ρ:-0.482; *p* = 0.008). No other significant associations between PUFAs and sarcopenia-defining parameters were found.

Total energy intake was found significantly and fairly correlated with handgrip strength (ρ:0.411; *p* = 0.027) and aLM (ρ:0.399; *p* = 0.032). Fat intake and MUFAs intake had inverse fair correlations with chair stand test time (ρ:-0.473; *p* = 0.011 for fat and ρ: -0.423; *p* = 0.025 for MUFA intake).

### Physical activity

**Fig. 2:**
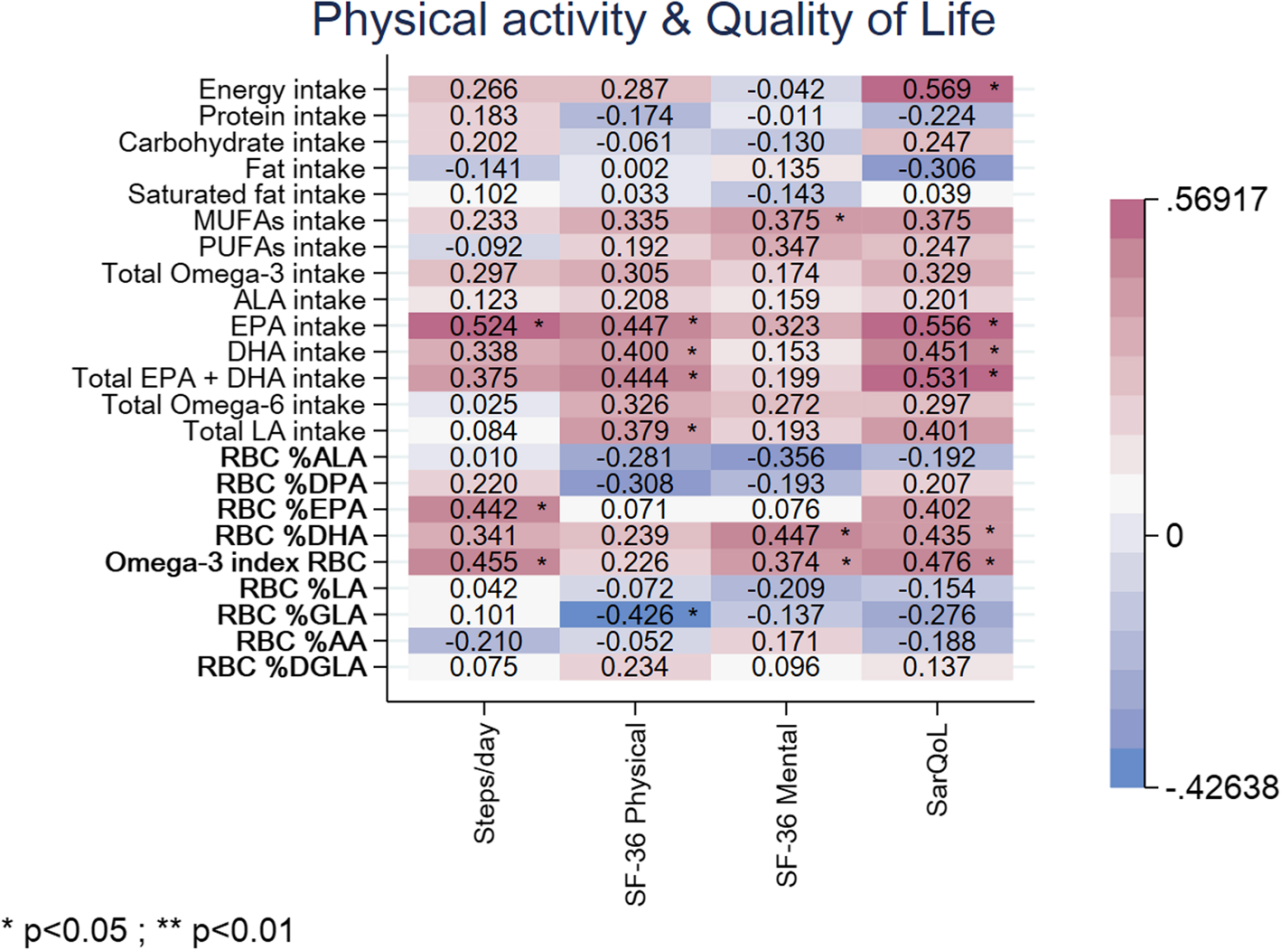
Heat plot representing correlation coefficients (ρ) between quality of life or physical activity parameters and PUFAs’ dietary intake and status. RBC = red blood cell; MUFAs = monounsaturated fatty acids; PUFAs = polyunsaturated fatty acids; LA = Linoleic acid; GLA = Gamma-linolenic acid; AA = Arachidonic acid; DGLA = Dihomo-gamma-linolenic; ALA = α-Linolenic acid; EPA = eicosapentaenoic acid; DPA = Docosapentaenoic acid; DHA = docosahexaenoic acid; SF-36 = Short Form 36 Health Survey

The participants in this study had a mean number of steps/day of 6879.6 ± 5721.5 through estimation with the physical activity monitor in *n* = 23 subjects. Both EPA intake (ρ:0.524; *p* = 0.010) as well as status (ρ:0.442; *p* = 0.035) had fair and significant correlations with step count (Fig. 2). Accordingly, the O3I in the RBC was also positively associated with step count (ρ:0.455; *p* = 0.029).

### Quality of life

SF-36 questionnaires were available in all (*n* = 29) participants of this study. Summary scores were calculated for the physical (mean 40.9 ± 8.4) and mental (mean 50.2 ± 10.2) components of this health questionnaire. The SF-36 physical component score was fairly associated with dietary intake of the omega-3 PUFAs EPA (ρ:0.447; *p* = 0.017), DHA (ρ:0.400; *p* = 0.035) and the sum of both (ρ:0.444; *p* = 0.018), see Fig. 2. However, this association could not be found for status of EPA, DHA or the O3I. Additionally, a fair positive association between SF-36 physical component score and dietary intake of omega-6 PUFA LA (ρ:0.379; *p* = 0.047) was found but not confirmed in the RBC status. In contrast, the SF-36 physical component score was inversely associated with the status of omega-6 GLA (ρ = -0.426; *p* = 0.024). Regarding the SF-36 mental component, the dietary intake of PUFAs was not significantly associated with the mental component summary scores. Fair positive correlations were found though with DHA status (ρ:0.447; *p* = 0.017) and the O3I (ρ:0.374; *p* = 0.049), as well as with dietary intake of MUFAs (ρ:0.375; *p* = 0.049).

Furthermore, overall score of the SarQoL questionnaire was calculated in *n* = 23 subjects with complete data (mean 61.6 ± 15.3). SarQoL score was found to be associated with intake of EPA (ρ:0.556; *p* = 0.006), DHA (ρ:0.451; *p* = 0.031) and the sum of both EPA and DHA intake (ρ:0.531; *p* = 0.009), as well as with the DHA status (ρ:0.435; *p* = 0.038) and the O3I in RBC (ρ:0.476; *p* = 0.022). Moreover, SarQoL score was found to be fairly associated with total energy intake (ρ:0.569; *p* = 0.005).

## Discussion

This study was the first to explore the relationship between PUFAs intake, status and sarcopenia outcomes in well-defined older adults with sarcopenia. The results suggested that omega-3 intake was below the recommended intake for this population. Moreover, there was no good correlation between dietary intake – assessed with 4-day food records – and PUFAs’ nutritional status in RBC. When exploring associations of PUFAs intake and status in this population, intake of EPA, DHA and LA were associated with indirect sarcopenia outcomes such as QoL and physical activity (EPA). Nutritional status of ALA (inversely) as well as DHA and its related O3I (positively) correlated with a direct sarcopenia-defining parameter aLM. Additionally, DHA and O3I status were positively associated with QoL measures (SF-36 mental score and SarQoL), whereas EPA and O3I status were associated with physical activity.

To our knowledge, no data on omega-3 *PUFAs intake* in sarcopenic older adults were published so far. As hypothesized, the mean total omega-3 intake, as well as EPA, DHA and ALA intake in our study population was below the dietary intake recommendations of the Belgian Superior Health Council [41]. Similarly, male omega-6 intake was also below the recommended intake. On the contrary, omega-6 intake of the women in this study was within the suggested range of 8.8–18 g/d.

When considering the *PUFAs status* in these older adults with sarcopenia, all RBC values were within the 99% confidence interval of the laboratory [47]. However, this gives us no information on the adequacy of the nutritional status, since no specific reference values to define optimal status are available yet.

In this population, PUFAs intake of EPA, DHA, ALA and LA did not correlate with PUFAs status in the RBC. The absence of a correlation between *dietary PUFAs intake and PUFAs status in RBC* immediately reflects the difficulty of adequately assessing the nutritional value of PUFAs in this population. Although 4-day food records already offer detailed insights on intake – certainly when days are spread over 2 weeks and the records carefully reviewed by the researcher together with the participant – it might be insufficient to represent long-term intake. Moreover, the high CV suggests that the interpersonal variation between participants was high and a longer recording of intake in a larger study sample might be required to assess detailed intake of PUFAs in this population. These 4-day food records should be considered a measure of short-term intake (measured over a period of 2 weeks), whereas the RBC biomarker might represent longer-term health status (past few months) [23].

Of the main *sarcopenia-defining parameters*, only associations of PUFAs status with aLM (muscle mass) and SPPB (physical performance) were found. Our data suggested an inverse association between ALA status and aLM, in contrast to a positive association of aLM with DHA status and O3I. Some factors might contribute to these seemingly contradictive findings. First, allthough EPA, DHA and ALA are all considered omega-3 PUFAs, there is a structural difference between EPA and DHA on the one hand and ALA on the other hand. The first two are considered ‘long-chain’ (≥ 20 carbon atoms) and considered responsible for a multitude of health benefits, whereas ALA is a ‘short-chain’ (precursor) omega-3 PUFA (≤ 18 carbon atoms) that acts a precursor of these PUFAs with a rather low conversion rate to long-chain omega-3 PUFAs in humans [48]. As such, less or no health benefits of ALA are expected. Second, recent data of NHANES suggested that protein intake also might influence the effect of omega-3 PUFAs on the muscle, by only finding a correlation between plasma omega-3 PUFAs and aLM in persons with low protein intake [49]. Regarding omega-6 PUFAs, an inverse association between AA status and SPPB score was found. This is consistent with the hypothesis that omega-6 PUFAs might promote inflammation and thereby reducing physical performance in older adults [50]. However, based on these exploratory data, it is too early to conclude which and how omega-6 PUFAs can achieve this.

*Physical activity* (step count) and EPA intake and status were positively associated, suggesting that subjects who ingested more EPA and improved their EPA status were more active. A recent systematic review could not confirm nor refute a potential association between EPA / DHA and physical activity in a variety of populations. However, it must be noted that most included studies were with young and athletic adults, rather than older adults [51].

*QoL* was associated with various parameters of PUFAs intake and status. When considering the more sarcopenia specific SarQoL questionnaire, EPA and DHA intake as well as DHA status were positively and fairly associated with overall SarQoL scores, suggesting that a diet rich in EPA and DHA might contribute to a better perceived physical QoL in these older adults. Previous research already suggested an association between SarQoL scores and nutritional status (assessed through the DETERMINE questionnaire) [52]. However, they did not investigate the specific association with PUFAs status. Regarding the SF-36, its physical component score was positively associated with both EPA and DHA intake, but not status. Furthermore, intake of the omega-6 PUFA LA was found positively associated, whereas GLA status was inversely associated with SF-36 physical component score. This is contrary to what was expected, as omega-6 PUFAs (like LA and GLA) tend to act pro-inflammatory in general. As such, one would expect that higher LA intake would decrease QoL in our patients, whereas GLA would be expected to improve QoL. However, the effect on inflammatory status of LA might be dependent on dosage, with lower intakes not having pro-inflammatory effects [53]. Furthermore, not all omega-6 PUFAs are pro-inflammatory, as is the case for GLA which is known to highly attenuate inflammation [54]. Regarding the SF-36 mental component summary score, a positive association with DHA status and O3I was found. These findings are supported by the results of *Noguchi *et al., who performed an intervention trial with DHA supplements in survivors of traumatic injury and found a positive correlation between change in RBC %DHA and SF-36 mental component scores after intervention [55]. To summarize, PUFAs intake and status might be intertwined with QoL but the exact relationship needs further research with both measures of PUFA’s intake and status.

When examining *energy intake* in present study, this is rather low. For older adults, an average intake of 30 kcal/kg body weight is recommended [56]. However, with a mean BMI of 25.3 ± 4.8 kg/m^2^ and weight of 70.98 ± 15.86 kg, the included participants tend to be overweight. As such, an average person with a weight of 70 kg would require around 2100 kcal/day intake, which is higher than the actual intake in the ENHANce participants (1894.73 ± 340.81 kcal/d). Main energy source in our participants were carbohydrates, followed by fat and protein. Still, total energy intake was positively associated with handgrip strength, aLM, and overall SarQoL score. Thus, emphasizing the importance of sufficient energy intake. Namely, it is accepted that the higher the energy intake is, the higher the chances of having a positive energy balance are (intake > expenditure), which may promote maintenance of muscle mass and strength with aging.

To the best of our knowledge, the present study was the first to determine this amount of detail on dietary intake and status of PUFAs in older adults with well-defined sarcopenia, according to the EWGSOP2. With the potential beneficial effects of PUFAs in mind, it is important to elucidate potential associations between its dietary intake, nutritional status and several sarcopenia outcomes, as has been done in present study. Another *strength* is the determination of not only intake but also PUFAs status. Moreover, the availability of data on all sarcopenia-defining parameters, as well as objectively measured physical activity and several QoL questionnaires add to the value of present study.

*A limitation* might be the number of days that food intake was recorded. As previously mentioned, the CV in the recorded intake of PUFAs suggested that 4 days were possibly too short to adequately assess dietary PUFAs intake. Nelson et al. suggested that 15–30 days of dietary records might be needed for an adequate calculation of individual PUFAs dietary intake [57]. Furthermore, with a small sample size of only 29 participants, this study aimed to be exploratory of nature and guide future research rather than make thorough conclusions on the complex PUFAs-sarcopenia relation. Intrinsically, the multitude of correlations tested in our small sample size carry a risk of type I statistical errors that cannot be excluded. Moreover, since the correlation between intake and status of PUFAs was poor, these results should be interpreted with caution.

## Conclusion

This exploratory study confirmed that omega-3 PUFAs intake was low in older adults with sarcopenia. Moreover, PUFAs intake and status did not correspond well in this population, suggesting that observing a good intake in food records does not necessarily correspond with an adequate PUFAs status. Furthermore, this study generated some interesting hypotheses regarding associations of PUFAs intake and status with sarcopenia outcomes, such as physical activity and QoL in older adults with sarcopenia. Unravelling the complex balance between beneficial and unfavourable PUFAs is challenging. In general, intake or status of omega-3 was positively associated with measures of sarcopenia, whereas intake of omega-6 was negatively associated. However, specific characteristics of the different kind of PUFAs (e.g., short-chain vs long-chain) and the duration of intake might play an important role in their health effects and require further clarification.

## Supplementary Information

**Additional file 1: Table S1.** STROBE-nut: An extension of the STROBE statement for nutritional epidemiology. **Table S2.** Schematic overview of results.

## Data Availability

The data that support the findings of this study are available on request from the corresponding author [JD]. The data are not publicly available due to them containing personal information of the participants that could compromise research participant privacy/consent.

## Abbreviations

4E-BP: Eukaryotic translation initiation factor 4E-binding protein
AA: Arachidonic acid
ALA: α-Linolenic acid
aLM: Appendicular lean mass
BMI: Body mass index
BW: Body weight
CST: Chair stand test
CV: Coefficient of variation
d: Day
DGLA: Dihomo-gamma-linolenic acid
DHA: Docosahexaenoic acid
dl: Deciliter
DPA: Docosapentaenoic acid
DXA: Dual-energy X-ray absorptiometry
E%: Energy %
e.g.: Exempli gratia
ENHANce: Exercise and Nutrition for Healthy AgeiNg study
EPA: Eicosapentaenoic acids
EWGSOP: European Working Group on Sarcopenia in Older People
FCDB: Food Composition Database
GLA: Gamma-linolenic acid
HGS: Handgrip strength
i.e.: Id est
IQR: Interquartile range
kcal: Kilocalorie
kg: Kilogram
L: Liter
m: Meter
mL: Milliliter
MMSE: Mini-Mental State Exam
mTOR: Mammalian target of rapamycin
MUFA: Monounsaturated fatty acid
n: Number
NCT: National Clinical Trial number
NEVO: Nederlands Voedingsstoffenbestand
ng: Nanogram
NUBEL: Nutriënten België
S6K1: P70S6 kinase
PUFA: Polyunsaturated fatty acid
QoL: Quality of life
RBC: Red blood cell
RCT: Randomized controlled trial
RDA: Recommended daily allowance
s: Second
SarQoL: Sarcopenia & Quality of Life
SD: Standard deviation
SF-36: 36-Item Short Form Health Survey
SMI: Skeletal muscle index
SPPB: Short Physical Performance Battery
STROBE: STrengthening the Reporting of OBservational studies in Epidemiology
USA: United States of America
y: Year
